# From an interventional study to a national scale-up: lessons learned from the Malakit strategy at the French Guiana–Suriname border

**DOI:** 10.1186/s12936-023-04672-6

**Published:** 2023-08-16

**Authors:** Maylis Douine, Hedley Cairo, Muriel Suzanne Galindo, Stephen Vreden, Yann Lambert, Antoine Adenis, Irene Jimeno, Martha Suarez-Mutis, Alice Sanna, Hélène Hiwat

**Affiliations:** 1 Inserm CIC 1424, Cayenne Hospital, Centre d’Investigation Clinique Antilles-Guyane, Cayenne, French Guiana; 2 TBIP, CNRS, Inserm, U1019-UMR9017-CIIL Centre d’Infection et d’Immunité de Lille, Institut Pasteur de Lille, Université de Guyane, Université de Lille, Cayenne, French Guiana; 3 National Malaria Elimination Programme, Ministry of Health of Suriname, Paramaribo, Suriname; 4 Foundation for the Advancement of Scientific Research, Paramaribo, Suriname; 5 Laboratory of Parasitic Diseases, Institute Oswaldo Cruz/Fiocruz, Rio de Janeiro, Brazil

**Keywords:** Scale-up, Malaria, Intervention, Elimination, Guiana Shield, Gold miners, Innovation, Amazon

## Abstract

Scaling-up an experimental intervention is always a challenge. On the border between French Guiana, Brazil and Suriname, an interventional study demonstrated the effectiveness of distributing self-diagnosis and self-treatment kits (Malakits) to control malaria in mobile and hard-to-reach populations. Its integration into the Suriname’s National Malaria Elimination Plan after a 2-year experiment faced numerous challenges, including human resources to cope with the additional workload of coordinators and to maintain the motivation of community health workers. The economic recession in Suriname, the Covid pandemic, and logistical issues also hampered the scale-up. Finally, thanks to the commitment of stakeholders in Suriname and French Guiana, the integration of Malakit distribution into the Surinamese national programme was proved possible.

## Background

Transforming access to diagnosis and treatment is fundamental to meeting the UN Sustainable Development Goals, especially the third one targeting to end malaria epidemics by 2030 [1]. Efficient innovations must be not stuck in the experimental stage but must be scaled up to improve the well-being of populations [2]. Scaling-up is defined as “deliberate efforts to increase the impact of health service innovations successfully tested in pilot or experimental projects to benefit more people and to foster policy and programme development on a lasting basis” [3]. To this end, strategic frameworks have been developed to address different aspects such as “Practical guidance for scaling-up health services innovations” from the World Health Organization (WHO) [3]. These guides address the question of advocacy, organization of scaling-up, financial and human resource aspects, governance and monitoring.

**Fig. 1:**
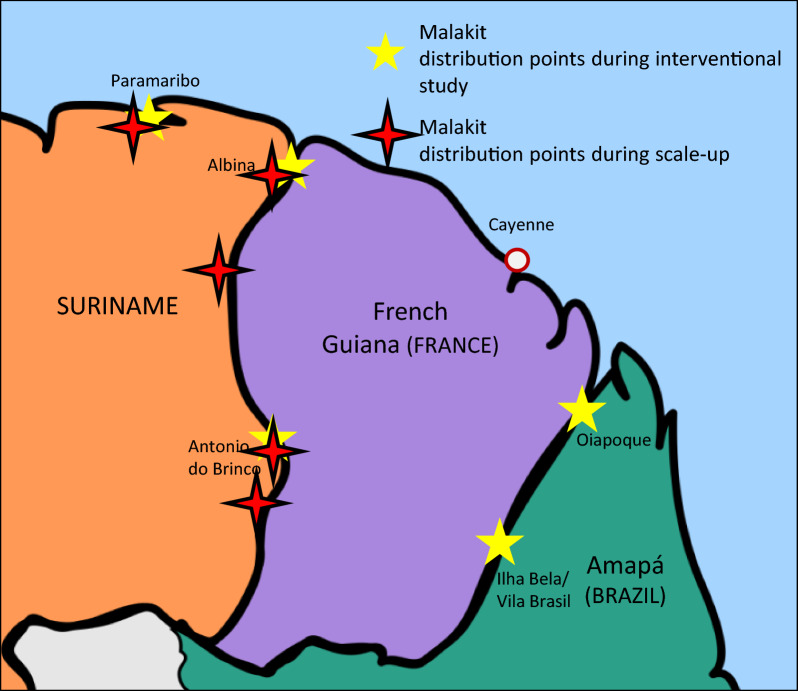
Map of Suriname–French Guiana–Amapá and the Malakit distribution during the interventional study and the scale-up

In the Guiana Shield, North-East South America and part of the Amazon biome, an interventional study has been implemented aiming at controlling malaria among the hard-to-reach population of workers in illegal gold mines spread on the French Guianese territory [4, 5]. Indeed, previous surveys have shown that this mobile population is highly affected by malaria and frequently use self-medication in the case of malaria symptoms [6–8]. The implemented strategy called Malakit is based on the distribution of self-diagnosis and self-treatment kits for malaria to this specific population in strategic French–Surinamese cross-border access points [4] (Fig. 1).

After a 2-years experimental period (2018–2020), the strategy has been integrated into the Surinamese National Malaria Elimination Programme (NMEP), the focus remaining on gold miners working in French Guiana. Through the example of the scaling up of the Malakit strategy, this article presents the success factors and challenges of scaling-up beyond the research phase.

There are already several guidelines for scaling up innovations. Nevertheless, each situation has its particularities and even if anticipation is very important, unpredictable events can occur. In the Malakit context, scaling-up has been concomitant with the Covid-19 pandemic and the economic crisis in Suriname. Thus, sharing experiences is an important input to address the challenges.

## Anticipating the transition to scale

The Malakit strategy was implemented from 2018 to 2020 as an interventional study at a geographical large scale aiming at including as much of the target population as possible (about 10,000 people). It evaluated the feasibility, acceptability and efficiency of this approach based on self-diagnosis and self-treatment for malaria.

As soon as the Malakit strategy was developed in 2017, the sustainability of the intervention was considered. As the target population, mainly from Brazil, is mobile between Brazil, French Guiana and Suriname, the health institutions of the three countries were involved [6]. Indeed, the distribution of the kits was implemented on the Surinamese and Brazilian territory as the distribution could not be performed on the French territory for regulatory reasons, so these two countries were on the front line to consider sustainability [4].

Several types of data were collected to allow the health authorities to decide on the relevance of the scale-up of the intervention: (i) effectiveness data (including kit use, impact on malaria epidemiology) [9, 10]; (ii) implementation and operational data [11]; (iii) qualitative data on the perception of the strategy by the population concerned [12]. Cost-effectiveness data would have been useful but were challenging to evaluate because of the very different economic contexts within the three countries.

## Learning by doing

Following a 2 years field experiment and results supporting the effectiveness of the strategy [9], the Surinamese Ministry of Health (MoH) decided in 2020 to upgrade the Malakit strategy from an interventional study to a national intervention in the NMEP [13]. Implementation of the strategy was established as part of a malaria grant supported by The Global Fund to fight AIDS, Tuberculosis and Malaria (TGFATM). The objective was to accelerate impact towards malaria elimination in Suriname (and the Guiana Shield Region) by addressing malaria in the cross-border moving populations.

The primary sponsor of the research study, the Centre Hospitalier de Cayenne (CHC) accompanied the further implementation of the strategy into the Surinamese NMEP because this translation process required adapting the shape of the intervention, the monitoring of indicators and staff training. The Regional Health Agency of French Guiana participated by funding a dedicated human resource in the CHC team. A Memorandum of Understanding (MoU) between CHC and Surinamese MoH formalized the creation of a steering committee and a monitoring-evaluation plan. Adaptations to the strategy were made to improve it in light of the experience gained during the experimental phase. One decision being to simplify data collection. Indeed, the data to be collected in the framework of scale-up do not need to be as exhaustive as for the research phase but are still necessary for the continued evaluation of the strategy itself.

A key point in scaling up is often the funding of long-term strategies after a pilot or experimental phase. Suriname's NMEP receives funding from TGFATM to support malaria elimination strategies. The Malakit strategy was successfully integrated into the application for the 2021–2024 TGFATM malaria grant. The use of WHO-certified tools (malaria rapid diagnostic test and anti-malarial treatment) in this kit was a condition for receiving Global Fund financing and thus facilitated the scaling up of the strategy. Indeed, innovations using new tools or treatments that are not certified by health institutions face specific challenges, such as regulatory issues that could be blocking the sustainability of innovations.

While obtaining this funding from TGFATM was a real opportunity, its continuation over time is not guaranteed and the amounts allocated to the Malakit distribution differ from the initial intervention research framework. The main task of NMEP’s community health workers (CHWs) is to screen people with symptoms for malaria, whether they are gold miners or not. With the integration of the Malakit strategy into NMEP's activities as a main intervention, they now have an additional task to perform, that of distributing kits and training kit users. The essential kit delivery training, however, is time-consuming and some CHWs consider that their salary is not sufficient for this additional workload. More so since their salaries were devaluated as a result of the recent economical downfall in Suriname. In addition, the repetition of training for gold miners can be wearying for CHWs, which leads to a loss of interest in this activity and, therefore, a loss of quality, or even resignation. Regular supervision of the teams in the field represents an opportunity for continuous training, recognition of the work accomplished and therefore for remotivation, but also considerable means in terms of human resources, time and transport, without additional funding. The high turnover, which is already a long-term challenge inherent to hiring CHWs from a mobile migrant population leads to an increased need for time dedicated to recruiting and training new CHWs.

This difficulty in sustaining CHW’s motivation may have also impacted that of gold miners to receive a kit. For the target population, which lives in numerous situations of vulnerability, health awareness is very different from population groups that have already met basic needs. Although the external qualitative study found that the target population was genuinely interested in receiving a kit [12], which was considered to meet a real need, a proactive approach by the CHWs to go and meet them, and to reinforce malaria and health education and propose a kit is essential. Especially since the decreased incidence of malaria at gold mining sites could lead to a decrease in interest in searching for a kit.

From a logistical perspective, the management (procurement, storage, and shipping) of the various components of the kit at the national level poses several challenges, which may result in stock-outs at some distribution sites. While specific resources were devoted to monitoring stocks and supervision during the research phase, the national coordination of these logistical aspects with equal resources by a team involved in several projects represents a considerable workload. Thus, integrating a new strategy into a national programme raises the importance of dedicated human resources (CHWs as well as supervisors and coordinators). This means finding competent and motivated people—which can sometimes be challenging—and fundings to pay salaries following the increased workload.

It should be noted that the transition between research and scale-up was concomitant with the Covid-19 pandemic, which has strongly impacted the health care system and saturated human resources.

All of the factors mentioned are thought to have played a role in the significant decrease in kit distribution after the end of the research phase. Efforts of the Surinamese NMEP supported by the CHC team are ongoing to engage new CHWs for the continuation of the strategy and to train them to distribute the Malakit. The coordination team remains in close contact with the CHWs to motivate them.

## Recommendations for the future

These factors are in line with the literature on scaling-up [14–16]. In the first place, several structural factors were limiting Malakit scale-up: availability of financial, material and human resources. The motivation of human resources could be reinforced in several ways: (1) salaries must be in line with the economic level to allow for a decent living; (2) recognition of the work accomplished by the hierarchy, through incentives or by offering good working conditions; (3) the increase of skills, through training and the diversification of tasks, which can be very repetitive over time; (4) recognition by peers of their role in society In this case, the economic crisis with the loss of purchasing power of mediators played a major role in the loss of motivation.

The outstanding factors (strategic plan for the scale-up, training and supervision) could be more easily addressed thanks to good anticipation and collaboration between the actors of the pilot phase and those of the scale-up [15]. The research aspect of the interventions with data monitoring and evaluation of the interventions facilitates the scale-up. The involvement of the communities is important, and this continues to be worked on in the Malakit intervention in a sustained manner, notably with qualitative studies on the perception of the health problem (i.e., malaria) but also of the intervention itself to adapt it to the needs of the communities.

Resources are sometimes easier to obtain for shorter innovation phases than for sustainable interventions, but the budget should not be underestimated as it is often found to be a limiting factor.

Stakeholder involvement is major. Scientific publications and advocacy with health institutions (national or international), funders and regulatory authorities are, therefore, important tools to support innovations [16]. In the present case, strong cooperation between health institutions, scientists and communities proved to be necessary and very effective.

## Conclusions

The transfer of the Malakit strategy to the Suriname NMEP proved possible despite a particularly challenging context. Difficulties persist in maintaining the quality of the intervention during scaling up. Significant financial and human efforts are needed and must involve institutions, researchers, funders, and the target population. Indeed, the gold miners targeted by the Malakit strategy, currently managed by the Surinamese NMEP, are mainly Brazilian people who work and contract malaria infection in French Guiana and other countries of the Guiana Shield.

This complex structure is the result of regulatory and geographical constraints that hinder intervention on the French territory. But to achieve malaria elimination at the Guiana Shield level, sub-regional collaboration is strongly needed. This also means that if action cannot be taken in one territory, that territory and its neighbouring should come together to design and implement alternative interventions. Malaria elimination will only be possible if efforts are sustained over time with a strong commitment from all stakeholders in the different countries involved.

## Data Availability

Not applicable.

## Abbreviations

CHC: Centre Hospitalier de Cayenne
CHW: Community health workers
MoU: Memorandum of Understanding
MoH: Ministry of Health
NMEP: National Malaria Elimination Program
TGFATM: The Global Fund to fight AIDS, Tuberculosis and Malaria
